# Supplementary material for: Cloning of nine glucocorticoid receptor isoforms from the slender African lungfish (Protopterus dolloi)
Source: PLoS One. 2022 Aug 1;17(8):e0272219. doi: 10.1371/journal.pone.0272219 (PMC9342798; doi:10.1371/journal.pone.0272219)
Supplement: Attachment — Submitted filename: LungfishGR-PLOSONE-RebuttaJuly12.docx [file pone.0272219.s001.docx]

Response to Reviewers.

We appreciate the constructive review of our manuscript “Cloning of Nine Glucocorticoid Receptor Isoforms from the Slender African lungfish (Protopterus dolloi)” PONE-D-22-09656. PLOS ONE

Below we describe our revisions to conform to the revisions requested by the academic editor and reviewer.

Academic Editor.

1. We have formatted headings to meet PLOS ONE's style requirements, including those for file naming.
2. Figures are submitted separately
3. Revised financial disclosure:

Reviewer’s comments.

1. Future functional characterization of nine isoforms will add great value to the current study.
**Response: Yes. We agree and are beginning to determine the EC50s for a panel of corticosteroids (cortisol, corticosterone, aldosterone, DOC, 11deoxycortisol, as well as dexamethasone.] This will take time as there are nine isoforms.**

2. Information on relative expression levels of these isoforms would be helpful to the readers.

**Response: Yes**, **and this also is a future study. It is important to know where and how strongly each GR is expressed.**

3. Can the authors make any inference about the reason why the lungfish has as many as nine isoforms? If so, please discuss.

**Response: At this time, the answer to this important question is a mystery to us. There are four GR isoforms in West African lungfish. Lungfish are mysterious.**
